# Methadone Maintenance Treatment, Sex Hormones, and Bone Mineral Density in Persons Who Inject Drugs

**DOI:** 10.1210/jendso/bvaf140

**Published:** 2025-09-01

**Authors:** Leen Wehbeh, Hsing-yu Hsu, Jenny Pena Dias, Kendall F Moseley, Susan Langan, Yutong Jiang, Damani A Piggott, Gregory D Kirk, Adrian S Dobs, Todd T Brown, Jing Sun

**Affiliations:** Division of Endocrinology, Diabetes, and Metabolism, Johns Hopkins University School of Medicine, Baltimore, MD 21287, USA; Bloomberg School of Public Health, Johns Hopkins University, Baltimore, MD 21287, USA; Division of Endocrinology, Diabetes, and Metabolism, Johns Hopkins University School of Medicine, Baltimore, MD 21287, USA; Division of Endocrinology, Diabetes, and Metabolism, Johns Hopkins University School of Medicine, Baltimore, MD 21287, USA; Bloomberg School of Public Health, Johns Hopkins University, Baltimore, MD 21287, USA; Bloomberg School of Public Health, Johns Hopkins University, Baltimore, MD 21287, USA; Division of Infectious Diseases, Johns Hopkins University School of Medicine, Baltimore, MD 21287, USA; Bloomberg School of Public Health, Johns Hopkins University, Baltimore, MD 21287, USA; Division of Infectious Diseases, Johns Hopkins University School of Medicine, Baltimore, MD 21287, USA; Division of Endocrinology, Diabetes, and Metabolism, Johns Hopkins University School of Medicine, Baltimore, MD 21287, USA; Division of Endocrinology, Diabetes, and Metabolism, Johns Hopkins University School of Medicine, Baltimore, MD 21287, USA; Bloomberg School of Public Health, Johns Hopkins University, Baltimore, MD 21287, USA

**Keywords:** methadone, bone mineral density, heroin, testosterone, estradiol

## Abstract

**Context:**

Methadone maintenance treatment (MMT) has been used to treat opioid use disorder, but is associated with bone loss. Sex hormones may mediate this relationship, though studies are limited.

**Objective:**

This work aimed to assess the relationships among bone mineral density (BMD) T-score, sex hormones, and MMT among individuals who inject drugs.

**Methods:**

We included study participants from the AIDS Linked to the Intravenous Experience (ALIVE) cohort. We performed multivariable linear regression to assess the association of MMT with BMD T-scores (lumbar spine, total hip, and femoral neck) in men and women. We further assessed whether sex hormones estradiol (E2) and free testosterone (free T) influenced the association between MMT and BMD in men and women. We controlled for age, sex, body mass index, smoking and heavy alcohol use (≥3 times per week), HIV status and/or hepatitis C virus viremia, vitamin D level, and heroin use.

**Results:**

Among 245 participants (153 men; 92 women), 107 were receiving MMT. Men undergoing MMT were more likely to have a low free T level compared to men not receiving MMT (odds ratio, 3.99; 95% CI, 1.40-11.39; *P* = .010). No significant differences in the odds of menopause in women by MMT status were observed. Individuals on MMT had significantly lower lumbar spine T-scores (β: −.55; 95% CI, −1.06 to −.05), independent of covariates. After adjusting for E2 and/or free T, the association between MMT and BMD was not significant, suggesting potential mediation.

**Conclusion:**

Among men who inject drugs, MMT may contribute to decreased BMD, potentially through decreased levels of free T and total E2.

The United States has experienced an unprecedented opioid epidemic, with rates of overdose-related death among the highest observed in US history [1, 2]. The Centers for Disease Control and Prevention recorded more than 100 000 drug overdose deaths in the United States in 2021, with overdose death rates continuing to rise steadily over the past two decades [2]. Since the 1960s, methadone maintenance treatment (MMT) has been used to help alleviate opioid withdrawal symptoms among people with opioid use disorder (OUD) [3]. Although MMT is effective in avoiding relapses, suppressing opioid cravings, and is considered safe when taken as prescribed, previous research has reported inhibitory effects of MMT on the hypothalamic-pituitary-gonadal axis [4‐8], including testosterone (T) deficiency and sexual dysfunction among men receiving MMT [4‐6, 9‐11]. Symptoms of hypogonadism such as infertility and loss of libido also have been reported both in men and women [11]. In addition to increased adverse endocrine events, MMT also is related to higher risk for low bone mineral density (BMD) [12, 13]. Hypogonadism is a risk factor for osteoporosis, with the reduction in bone mass leading to increased fractures, worsened quality of life, and increased comorbidities [14].

Despite evidence showing opioids’ effects on the endocrine system, systematic reviews have shown inconsistent results relative to methadone's effect on the hypothalamic-pituitary-gonadal axis, when assessing different methadone dosage and length of use [7, 15, 16]. Study sample size, study design, and variables taken into account were also considered to be reasons for the discrepancies. A recent Endocrine Society Scientific Statement identified major scientific gaps in research associated with the effect and clinical consequences of opioids on the endocrine system [16]. To our knowledge, few studies have assessed the complex interplay of MMT, hormones, and BMD among men and women with OUD. The objective of the present study is to investigate the differences in BMD and sex hormones among people with a history of injection drug use who are receiving or not receiving MMT. Our hypothesis is that there are significant differences in BMD and these differences are moderated through sex hormones among men and women who use substances.

## Materials and Methods

### Study Population and Data Collection

This is a cross-sectional study nested within the AIDS Linked to the IntraVenous Experience (ALIVE) cohort. The ALIVE cohort has prospectively followed adults with a history of injection drug use in a community-recruited cohort on a semiannual basis since 1988 [17]. For this substudy, we recruited 4 groups of ALIVE participants who have been exposed to HCV (HCV antibody positive) and/or HIV (HCV viremia/HIV–, HCV aviremia/HIV–, HCV viremia/HIV+, HCV aviremia/HIV+) between March 2016 and December 2019. Because the ALIVE cohort predominately consists of adults with African American ancestry, we included only participants with African American ancestry in the present study. We excluded individuals with end-stage renal disease or those who had received successful treatment for HCV at study baseline. The study was approved by the Johns Hopkins Institutional Review Board, and all participants provided written informed consent.

At semiannual visits, ALIVE participants completed standardized questionnaires and underwent clinical examination. Detailed information obtained at each follow-up visit included socioeconomic, behavioral, and clinical parameters for the prior 6-month period. Smoking and substance use, including alcohol, tobacco and illicit injection and noninjection drug use, were assessed by participant self-report of behaviors in the prior 6-month period. Methadone and heroin use were assessed by participant self-report in the prior 6-month period. Comorbid conditions ascertained included participant self-report of any provider diagnosis of diabetes, hypertension, stroke, cardiovascular, renal, and chronic lung disease. Hazardous alcohol use (≥3 times per week) was assessed using the Alcohol Use Disorders Identification Test (AUDIT) [18]. Menopausal status for women was assessed based on self-report and the date of last menstrual cycle (menopause was assigned if last menstrual cycle was > 12 months earlier).

### Hepatitis C Virus, HIV, and Methadone Use Classification

All participants were exposed to HCV (HCV antibody positive). CD4+ cell counts were measured by standard flow cytometry using Multitest CD3/CD8/CD45/CD4W/Trucount CE reagent (BD Biosciences, catalog No. 342447, Research Resource Identifier [RRID]: AB_2868792). HIV-1 plasma RNA levels were assessed by COBAS AmpliPrep/COBAS TaqMan HIV-1 monitor with a lower limit of detection of 50 copies/mL (Roche Diagnostics). HCV RNA plasma quantification was performed at the Johns Hopkins Pathology laboratory by COBAS 6800/8800 systems (Roche Diagnostics) with a lower limit of detection of 15 copies/mL, and HCV immunoglobulin G antibody testing was conducted as previously described.

HCV status was classified in this study based on the presence or absence of HCV viremia (HCV RNA positive). HIV status was classified in this study into 3 categories: 1) HIV negative, 2) HIV positive with undetectable HIV viral load, and 3) HIV positive with detectable HIV viral load.

For current MMT, study participants were categorized into 2 groups based on self-reported use at the time of the substudy visit: 1) no MMT and 2) MMT.

### Biochemical Measurements

#### Sex hormones and hypogonadism/menopause status

Phlebotomy was performed before noon. Samples were frozen at −80 °C until the time of measurement. T, estradiol (E2), and sex hormone–binding globulin (SHBG) were measured at the Brigham Research Assay Core Laboratory at Brigham and Women's Hospital, Boston, Massachusetts. T and E2 were extracted by solid phase extraction and eluted by high-performance liquid chromatography. Quantification of sex hormones was performed by mass spectrometry (MS) in electrospray ionization source. Deuterated stable isotope was used for the calibration of the assay. The T assay had a sensitivity of 1 ng/dL, with an interassay coefficient of variation (CV) (male < 7%, female < 5%) and intra-assay CV (male < 2%, female < 5%). The E2 assay had a sensitivity of 1 ng/mL with an interassay CV < 12%, and intra-assay CV < 5%. SHBG concentrations were measured using a chemiluminescent assay (Beckman Coulter) with an interassay CV between 5.2% and 5.5%, intra-assay CV between 4.5% and 4.8%, and sensitivity of 0.33 nmol/L. Free T levels were measured by equilibrium dialysis [19, 20]. Given the high prevalence of abnormalities in SHBG in our population, we assessed gonadal status with free T, rather than total, and defined free T less than 50 pg/mL as biochemical hypogonadism.

### Vitamin D Levels

Vitamin D levels were determined using liquid chromatography–tandem MS. 25-Hydroxyvitamin D3 and D2 in serum were extracted by solid phase extraction, separated and eluted by high-performance liquid chromatography, and determined by MS in atmospheric-pressure chemical ionization source at positive ionization mode and multiple reaction monitoring of transition.

### Bone Composition and Bone Mineral Density Measurements

Body mass index (BMI: calculated as kg/m^2^) was calculated for all participants. Dual-energy x-ray absorptiometry (DXA) scans were performed on a Hologic Horizon machine (Hologic Inc) to obtain measures of BMD at the lumbar spine (LS), total hip (TH), and femoral neck (FN). T-scores were calculated from site-specific BMD measures using the White, premenopausal female database as a standard reference population for T-scores per International Society for Clinical Densitometry recommendations [21].

### Statistical Analysis

Baseline demographic and biochemical measures of the study participants were described and stratified based on sex and methadone use, and normality of the distributions for each variable was checked by plotting histograms. We used multivariable logistic regression to assess the relationship between methadone use and low T in men and postmenopausal status in women adjusted for multiple variables including age, BMI, smoking, alcohol use, HIV status (HIV negative, HIV positive with undetectable viral load, and HIV positive with detectable viral load), HCV viremia (all participants are HCV antibody positive or have been exposed to HCV), methadone use, and heroin use. We additionally adjusted for heroin use, which is a major confounder in the relationship between methadone use and BMD. We then assessed the relationship between methadone use and E2 using multiple linear regression, adjusted for the previous variables and free T. We also examined the association between site-specific BMD T-scores and methadone use using multiple linear regression, adjusting for the aforementioned variables in addition to serum levels of vitamin D, free T, and E2 separately and together in the same model. Covariates were selected based on previous literature, the study hypothesis, and the effect sizes in the preliminary univariate analyses. To assess the robustness of our findings to potential unmeasured confounding, we conducted a sensitivity analysis using an omitted variable bias framework for our primary associations of interest. Stata version 18 was used for all statistical analyses.

## Results

### Study Population

The study population consisted of 245 participants, 153 men and 92 women. Of the men, 54 (35.3%) were receiving MMT and 98 were not. Of the women, 53 (57.6%) were receiving MMT and 39 were not. Table 1 presents the demographic characteristics stratified by sex and methadone use. Among men, those receiving MMT were more likely to be current smokers or heroin users. Those receiving MMT also showed lower total T, free T, and E2 levels. Among women, those receiving MMT were more likely to be current smokers, heroin users, and alcohol users. However, T and E2 levels were similar regardless of MMT use.

**Table 1.** Baseline characteristics of the AIDS Linked to the Intravenous Experience study participants, by sex and methadone use

Variables	Men (N = 153)			Women (N = 92)		
	No methadone (N = 99)	Methadone use (N = 54)	*P^a^*	No methadone (N = 39)	Methadone use (N = 53)	*P^a^*
Age, mean (IQR), y	58 (54-63)	56.8 (53-61)	.30	54 (50-59)	54 (50-57)	.93
BMI category			.52			.87
Normal weight (18.5 ≤ BMI < 25)	55 (56%)	30 (56%)		10 (26%)	16 (30%)	
Overweight (25 ≤ BMI < 30)	30 (30%)	13 (24%)		11 (28%)	15 (28%)	
Obese (BMI > 30)	14 (14%)	11 (20%)		18 (46%)	22 (42%)	
Education			.69			.23
≤High school	65 (66%)	36 (67%)		24 (62%)	39 (74%)	
Some college/technical school	24 (24%)	15 (28%)		10 (26%)	12 (23%)	
Associate, bachelor's or master's degree	9 (9%)	3 (5%)		5 (13%)	2 (4%)	
Unknown	1 (1%)	0 (0%)				
Alcohol*^b^*	8 (8%)	5 (9%)	.80	0 (0%)	5 (10%)	.049
Smoking*^c^*	59 (60%)	46 (85%)	.001	18 (46%)	37 (70%)	.022
Heroin use*^d^*	41 (42%)	32 (60%)	.034	9 (24%)	27 (53%)	.005
HCV, viremia	69 (70%)	43 (80%)	.18	23 (59%)	34 (64%)	.61
HIV			.13			.038
HIV–	65 (66%)	39 (72%)		20 (51%)	38 (72%)	
HIV+/undetectable VL	23 (23%)	14 (26%)		14 (36%)	8 (15%)	
HIV+/detectable VL	11 (11%)	1 (2%)		5 (13%)	7 (13%)	
**Vitamin D3, ng/mL**			.031			.60
≤10	24 (24%)	5 (9%)		10 (26%)	9 (17%)	
10-20	40 (40%)	32 (59%)		13 (33%)	20 (38%)	
>20	35 (35%)	17 (31%)		16 (41%)	24 (45%)	
Total testosterone, ng/dL, median (IQR)	509 (319-668)	339 (244-454)	<.001	18.8 (12.2-33.0)	19.6 (13.9-26.3)	.75
Free testosterone, pg/mL, median (IQR)	117 (73 168)	66 (47-82)	<.001	5.4 (4.4-8.0)	6.1 (4.3-8.1)	.75
Estradiol, pg/mL, median (IQR)	13 (7-20)	9 (6-13)	.009	4.0 (2.2-8.9)	3.5 (2.4-5.8)	.68
Menopause	—	—	—	25 (64%)	34 (64%)	≥.999

Abbreviations: BMI, body mass index, HCV, hepatitis C virus; IQR, interquartile range.

^a^ *P* values are tested by chi-square test for categorical variables and log-rank test for continuous variables.

^b^Alcohol: average 3 or more times per week.

^c^Smoking: current cigarette smoking (yes or no).

^d^Heroin use: any heroin in the past 6 months.

The median age of the cohort was 56 years (interquartile range [IQR]: 51-61 years), and the mean BMI was 27 kg/m^2^ (IQR: 22-30). Seven percent of participants were alcohol users (>3 drinks/week), and 65% were current smokers. All participants were exposed to HCV in the past and had positive HCV antibody, but 69% had detectable HCV viremia. Eighty-one participants (33%) were people living with HIV, and, of those, 58 had undetectable HIV viral load and 23 had detectable HIV viral load.

Table 2 presents participant BMD, stratified by sex and methadone use. Among men, those that were using methadone had lower lumbar spine BMD (median: 1.005 vs 1.094 g/cm^2^; *P* = .013) and lumbar spine T-score (median: −0.4 vs 0.4; *P* = .019) compared to those not using methadone. BMD and T-scores at the hip and FN were similar by methadone use status. No difference in BMD or T-score at any of the sites was observed among the women.

**Table 2.** Baseline bone mineral density of the AIDS Linked to the Intravenous Experience study participants, by sex and methadone use

Variables	Men (N = 153)			Women (N = 92)		
	No methadone (N = 99)	Methadone use (N = 54)	*P^a^*	No methadone (N = 39)	Methadone use (N = 53)	*P^a^*
LS BMD (g/cm^2^), median (IQR)	1.094 (0.972 to 1.216)	1.005 (0.891 to 1.127)	.013	1.041 (0.841 to 1.136)	0.994 (0.911 to 1.119)	.34
FN BMD (g/cm^3^), median (IQR)	0.871 (0.799 to 0.978)	0.875 (0.754 to 0.988)	.72	0.824 (0.731 to 0.936)	0.804 (0.712 to 0.901)	.36
TH BMD (g/cm^3^), median (IQR)	1.004 (0.920 to 1.123)	0.988 (0.857 to 1.097)	.24	0.951 (0.848 to 1.055)	0.942 (0.825 to 1.023)	.66
LS T-score (pg/mL), median (IQR)	0.4 (−0.7 to 1.5)	−0.4 (−1.4 to 0.8)	.019	−0.1(−1.9 to 0.8)	−0.5 (−1.3 to 0.7)	.55
FN T-score (pg/mL), median (IQR)	0.2 (−0.4 to 1.2)	0.2 (−0.9 to 1.2)	.72	−0.2 (−1.2 to 0.8)	−0.4 (−1.2 to 0.5)	.58
TH T-score (pg/mL), median (IQR)	0.5 (−0.2 to 1.5)	0.4 (−0.7 to 1.3)	.24	0.075 (−0.613 to 0.675)	0.002 (−0.956 to 0.660)	.73
Spine BMD category			.14			.57
Normal BMD (T-score > −1)	77 (78%)	35 (65%)		27 (69%)	35 (65%)	
Osteopenia (T-score −1 to −2.4)	19 (19%)	18 (33%)		10 (26%)	12 (23%)	
Osteoporosis (T-score ≤ −2.5)	3 (3%)	1 (2%)		2 (5%)	6 (11%)	
Low BMD at any site*^b^*	28 (29%)	23 (43%)	.073	14 (36%)	23 (43%)	.47

Abbreviations: BMD, bone mineral density as represented by T-score; FN, femoral neck; IQR, interquartile range; LS, lumbar spine; TH, total hip.

^a^ *P* values are tested by chi-square test for categorical variables and log-rank test for continuous variables.

^b^Low BMD: defined as T-score less than or equal to −1 at any of the 3 sites (LS, FN, or TH).

### Methadone Use, Low Free Testosterone, and Menopause

Table 3 presents the relationship between methadone use, low free T, and E2 in men and the relationship between methadone use and menopause status in women in adjusted models. Methadone use (odds ratio [OR] 3.99; 95% CI, 1.40-11.39; *P* = .010) and heroin use (OR 7.66; 95% CI, 2.27-25.91; *P* = .001) were both significantly associated with higher odds of low free T in men. Similarly, a BMI greater than 30 kg/m^2^ (compared to normal BMI) (OR 7.00; 95% CI, 1.76-27.90; *P* = .006) was associated with higher odds of low free T in men. In addition, methadone use was also significantly associated with low E2 among men (−3.93 pg/mL; 95% CI, −7.30 to −0.56; *P* = .023), but this was not statistically significant after additional adjustment for free T. Heroin use was associated with lower E2 in men (−5.23 pg/mL; 95% CI, −8.49 to −1.97; *P* = .002), which remained statistically significant after further adjustment for free T (*P* = .026). Free T was associated with lower E2 (0.05; 95% CI, 0.03-0.08; *P* < .001). We did not observe statistically significant differences in the odds of menopause in women with methadone (OR 2.74; 95% CI, 0.35-21.41; *P* = .338) or heroin use (OR 0.52; 95% CI, 0.07-3.96; *P* = .531). As expected, there were significantly higher odds of menopause with each 1-year increase in age (OR 2.97; 95% CI, 1.58-5.58; *P* = .001). Given the collinearity with age, when we removed age from the model, methadone use (OR 1.70; 95% CI, 0.58-4.99; *P* = .337) was also not associated with menopause. However, the relationship between heroin use and menopause became significant (OR 0.30; 95% CI, 0.10-0.92; *P* = .035).

**Table 3.** Association of methadone use with low free testosterone and low estradiol in men and menopause in women in the AIDS Linked to the Intravenous Experience cohort

	Low free T in men*^c^*			Estradiol in men*^d^* (model 1)			Estradiol in men (model 1 + free T)			Menopause in women*^e^*		
	OR	95% CI	*P*	β	95% CI	*P*	β	95% CI	*P*	OR	95% CI	*P*
Methadone	3.99	1.40 to 11.39	.010	−3.93	−7.30 to −.56	.023	−1.00	−4.54 to 2.55	.580	2.74	0.35 to 21.41	.338
Age	1.09	0.99 to 1.20	.079	−.27	−0.53 to −.02	.037	−.21	−0.46 to 0.03	.090	2.97	1.58 to 5.58	.001
BMI*^a^*												
Normal weight	Ref.			Ref.			Ref.			Ref.		
Overweight	0.74	0.20 to 2.67	.644	−.74	−4.32 to 2.84	.684	−.29	−3.72 to 3.13	.865	1.84	0.15 to 21.85	.630
Obese	7.00	1.76 to 27.90	.006	3.01	−1.57 to 7.60	.196	3.82	−0.57 to 8.20	.088	0.26	0.02 to 2.83	.268
Smoking	1.50	0.45 to 5.00	.507	−.38	−3.92 to 3.15	.830	.04	−3.33 to 3.41	.982	3.73	0.49 to 28.30	.203
Alcohol*^b^*	4.52	1.07 to 19.09	.040	−2.64	−8.07 to 2.79	.339	−1.18	−6.41 to 4.05	.657	0.47	0.01 to 27.68	.714
HIV												
HIV–	Ref.			Ref.			Ref.			Ref.		
HIV+/undetectable	1.02	0.31 to 3.37	.968	6.16	2.60 to 9.73	.001	5.37	1.95 to 8.79	.002	0.70	0.07 to 7.03	.760
HIV+/detectable	5.60	0.95 to 32.99	.057	−5.28	−11.27 to 0.71	.084	−3.87	−9.62 to 1.88	.185	0.42	0.003 to 6.77	.729
HCV viremia	0.70	0.21 to 2.36	.570	1.01	−2.59 to 4.61	.579	.99	−2.44 to 4.41	.570	0.74	0.09 to 6.21	.784
Heroin	7.66	2.27 to 25.91	.001	−5.23	−8.49 to −1.97	.002	−3.65	−6.86 to −0.44	.026	0.52	0.07 to 3.96	.531
Free T, pg/mL	—	—	—	—	—	—	.05	0.03 to 0.08	<.001	—	—	—

Abbreviations: BMI, body mass index; HCV, hepatitis C virus; OR, odds ratio; Ref., reference; T, testosterone.

^a^BMI: normal weight: 18.5 less than or equal to BMI less than 25; overweight: 25 less than or equal to BMI less than 30; obese: BMI greater than 30.

^b^Alcohol: average 3 or more times per week.

^c^Low free T defined as free T less than 50 pg/mL.

^d^Estradiol as continuous outcome.

^e^Menopause defined as self-report of last menstrual period more than 12 months prior.

### Association of Methadone Use and Bone Mineral Density

**Figure 1.**
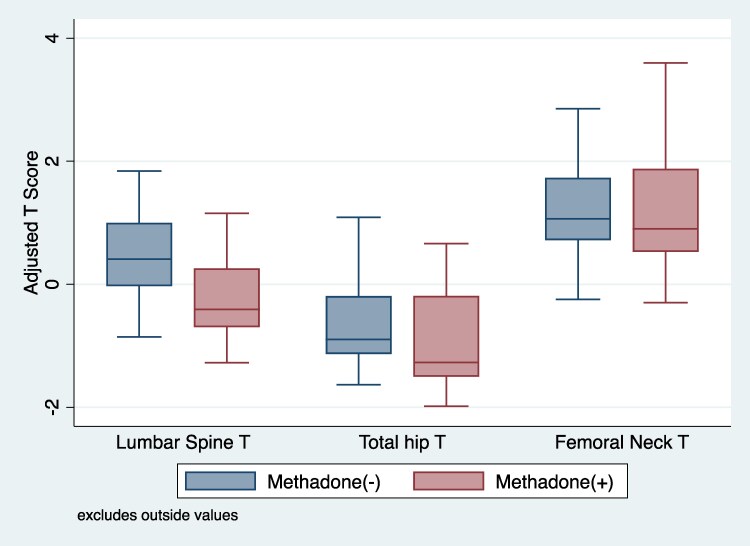
Marginal prediction of site-specific bone mineral density by methadone use in men in the ALIVE cohort. T-scores were adjusted for age, body mass index (normal weight vs overweight vs obese), smoking status (yes vs no), alcohol use (yes vs no), HIV status (HIV– vs HIV+/undetectable vs HIV+/detectable), hepatitis C virus status (yes vs no), vitamin D (>20 ng/mL vs 10-20 ng/mL vs < 10 ng/mL), methadone use (yes vs no), and heroin use (yes vs no).

**Figure 2.**
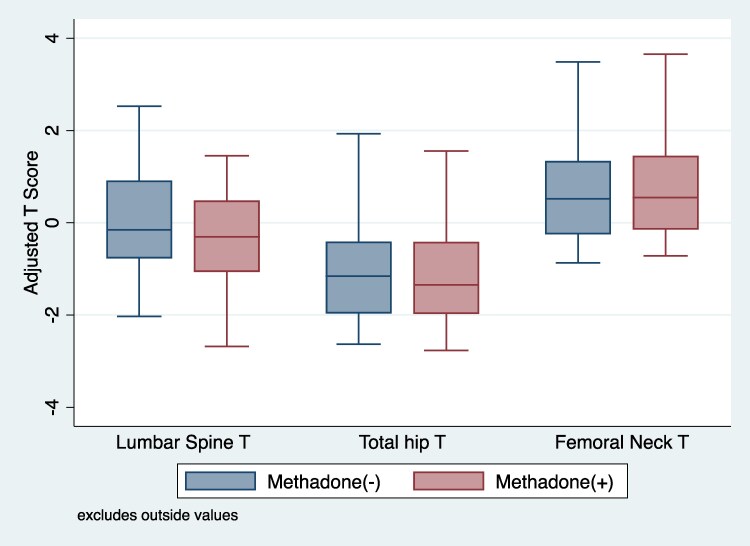
Marginal prediction of site-specific bone mineral density by methadone use in women in the ALIVE cohort. T-scores were adjusted for age, body mass index (normal weight vs overweight vs obese), smoking status (yes vs no), alcohol use (yes vs no), HIV status (HIV– vs HIV+/undetectable vs HIV+/detectable), hepatitis C virus status (yes vs no), vitamin D (>20 ng/mL vs 10-20 ng/mL vs < 10 ng/mL), menopause status (yes vs no), methadone use (yes vs no), and heroin use (yes vs no).

We observed that methadone use was associated with significantly lower lumbar spine T-score in men (−0.55; 95% CI, −1.06 to −0.05; *P* = .032; Table 4). This association was independent of age, BMI, alcohol use, HIV and HCV status, and vitamin D. There was no significant association between heroin use (−0.07; 95% CI, −0.56 to 0.43; *P* = .794) and lumbar spine T-score. The robustness value for methadone use is 0.13, indicating that an unmeasured confounder would need to explain 13.5% of the residual variance to eliminate the observed −0.55 difference in BMD T-scores among methadone users. This suggests that the estimated association is reasonably robust to unmeasured confounding in our present model. We did not observe a significant association between methadone use and BMD in TH and FN BMD in men and women (Supplementary Tables S1-S3). However, consistent with the model in LS, we observed the BMD T-scores in TH and FN were lower in those receiving MMT compared to those not receiving MMT, albeit not statistically significantly so (Fig. 1 and Fig. 2).

**Table 4.** Association between methadone use and lumbar spine bone mineral density T-score in men and women in the AIDS Linked to the Intravenous Experience cohort

	β	95% CI	*P*
Methadone	−.55	−1.06 to −.05	.032
Age	−.03	−0.07 to 0.01	.173
Female	−.77	−1.31 to −.24	.004
BMI*^a^*			
Normal weight	Ref.		
Overweight	.92	0.36 to 1.48	.001
Obese	1.19	0.56 to 1.83	<.001
Smoking*^b^*	0.50	−0.02 to 1.02	.061
Alcohol*^c^*	−0.35	−1.23 to 0.53	.439
HIV			
HIV–	Ref.		
HIV+/undetectable	−0.42	−0.97 to 0.13	.136
HIV+/detectable	−0.04	−0.86 to 0.78	.917
HCV viremic	0.20	−0.31 to 0.72	.438
Vitamin D, ng/mL			
>20	Ref.		
10-20	−0.04	−0.56 to 0.48	.884
<10	−0.21	−0.85 to 0.43	.522
Heroin*^d^*	−0.07	−0.56 to 0.43	.794

Abbreviations: BMI, body mass index; HCV, hepatitis C virus; Ref., reference.

^a^BMI: normal weight: 18.5 less than or equal to BMI less than 25; overweight: 25 less than or equal to BMI less than 30; obese: BMI greater than 30.

^b^Smoking: current cigarette smoking (yes or no).

^c^Alcohol: average 3 or more times per week.

^d^Heroin use: any heroin in the past 6 months.

### Role of Sex Hormones in the Association Between Methadone Use and Lumbar Spine T-Score

We conducted further analyses among the men to assess whether the relationship between MMT and lower LS T-score could be explained by alterations in sex hormones. Table 5 presents the relationship between methadone use, sex hormones, and LS T-score in men. In multiple linear regression analysis adjusted for multiple variables (age, BMI, alcohol use, HIV and HCV status, vitamin D, methadone, and heroin), methadone, but not heroin use, was associated with significantly lower T-score (−0.67; 95% CI, −1.31 to −.02; *P* = .044) (model 1). When E2 was added to the model (model 2), the association between methadone use and LS T-score was no longer statistically significant and the point estimate decreased by 35% (−0.44; 95% CI, −1.10 to 0.22; *P* = .186); higher E2 was associated with higher LS T-score (0.05; 95% CI, 0.01-0.08; *P* = .005). When free T was added to the model instead of E2 (model 3), the association between methadone and LS T-score was also no longer statistically significant and the point estimate decreased by 67% (−0.22; 95% CI, −0.93 to 0.49; *P* = .541); higher free T was associated with higher LS T-score (0.01; 95% CI, 0.002-0.01; *P* = .006). When both E2 and free T were added to the model together (model 4), the association between methadone and T-score was no longer statistically significant (−0.18; 95% CI, −0.88 to 0.52; *P* = .619); however, both E2 and free T were independently associated with higher LS T-score (0.04; 95% CI, 0.003-0.07; *P* = .035) and (0.01; 95% CI, 0.0001-0.01; *P* = .045).

**Table 5.** Association of methadone use with lumbar spine bone mineral density T score and sex hormones in men in the AIDS Linked to the Intravenous Experience cohort

	Model 1		Model 2		Model 3		Model 4	
	β	*P*	β	*P*	β	*P*	β	*P*
Methadone	−.67	.044	−.44	.186	−.22	.541	−.18	.619
Age	−.014	.583	.002	.945	−.002	.934	.006	.809
BMI*^a^*								
Normal weight	Ref.		Ref.		Ref.		Ref.	
Overweight	1.03	.003	1.07	.002	1.10	.002	1.11	.001
Obese	.84	.062	.73	.103	.98	.030	.85	.057
Smoking*^b^*	.36	.289	.40	.234	.43	.203	.44	.192
Alcohol*^c^*	−.16	.754	−.04	.934	.03	.949	.08	.882
HIV								
HIV–	Ref.		Ref.		Ref.		Ref.	
HIV+/undetectable	−.06	.867	−.34	.336	−.16	.641	−.36	.308
HIV+/detectable	.13	.827	.39	.497	.36	.534	.49	.389
HCV viremic	.36	.308	.29	.402	.33	.342	.29	.399
Vitamin D, ng/mL								
>20	Ref.		Ref.		Ref.		Ref.	
10-20	0.43	.199	0.42	.207	0.51	.124	0.48	.142
<10	0.15	.733	0.20	.643	0.24	.578	0.25	.533
Heroin*^d^*	−0.51	.104	−0.24	.463	−0.25	.442	−0.12	.710
Estradiol, pg/mL	−	−	0.05	.005	−	−	.04	.035
Free T, pg/mL	−	−	−	−	.01	.006	.01	.045

Abbreviations: BMI, body mass index; HCV, hepatitis C virus; Ref., reference; T, testosterone.

^a^BMI: normal weight: 18.5 less than or equal to BMI less than 25; overweight: 25 less than or equal to BMI less than 30; obese: BMI greater than 30.

^b^Smoking: current cigarette smoking (yes or no).

^c^Alcohol: average 3 or more times per week.

^d^Heroin use: any heroin in the past 6 months.

Supplementary Table S4 presents the relationship between methadone use and LS T-score in women. We did not observe a significant relationship between methadone use and LS T-score among women, whether adjusted for E2, free T, or both.

## Discussion

Using comprehensive measurements of methadone maintenance, BMD, as well as sex hormones from the ALIVE cohort, we observed a significant independent association between methadone use and lower LS T-score in men with a history of injection drug use. Methadone use was significantly associated with lower free T and total E2 among men. Furthermore, serum levels of both E2 and free T potentially mediated the association between methadone use and LS T-score, highlighting a probable underlying mechanism. While we did not observe a significant association between methadone use or heroin on menopause among women, BMD was lower among women with methadone use. This study provides further evidence of the effect of methadone use on BMD potentially through the pathway of sex hormones in men and women with a history of substance use.

Our findings of the adverse effect of methadone use on the hypothalamic-pituitary-gonadal axis among men was consistent with previous studies [4, 16, 22‐25]. This effect results from the direct action on opioid receptors in the hypothalamus and the inhibition of gonadotropin-releasing hormone secretion due to the stimulatory effect on prolactin release [25‐29]. Previous research showed inconsistent results for the association between methadone, heroin, and BMD T-scores [30‐33]. Arnsten et al [30, 31] observed only lowered LS T-scores but not TH or FN T-scores among men and women receiving methadone. Results from Grey et al [32] showed lower T-scores in all 3 sites among men, but not women. Sharma et al [33] reported lowered LS T-score, but not TH or FN T-scores among women. The discrepancy could stem from the differences between study populations. Grey et al [32] focused on middle-aged individuals that have been receiving MMT for several years, and while both focused on middle-aged women only, Sharma et al [33] examined current and previous methadone use and Arnsten et al [30, 31] current methadone use. Our study aimed to assess the relationship of current methadone use to BMD, and there might be different results when evaluating cumulative use or long-term use of methadone.

The magnitude of the effect of methadone maintenance therapy that we observed in LS BMD in men was approximately 0.5 SDs, which may be clinically significant. In a meta-analysis in the general population, 1 SD lower LS BMD was associated with a 50% increase in osteoporotic fracture [34]. While relatively few individuals in our cohort had BMD in the osteoporotic range, the average age was 54 years and approximately one-third had BMD in the low BMD range, using the young White female reference database [35]. These results suggest that these individuals receiving MMT may be at risk for future bone loss and fracture, and DXA screening may be warranted at an earlier age than current guidelines [36].

Among men in the cohort, methadone maintenance therapy was associated with lower free T and lower total E2 concentrations. While the effect of opioid therapy on gonadal function has been well documented [10], fewer studies have examined the effects of methadone maintenance therapy on E2 concentrations in men. In a sample of 54 men receiving chronic opioids (24 receiving methadone), there was a dose-dependent decrease in serum E2, which was approximately 50% lower than the reference population [23]. Similarly, among men, methadone maintenance therapy and recent heroin use were independently and significantly associated with lower total E2 concentrations. While decreased E2 concentrations may have been related to lower substrate availability to convert T to E2 via aromatase (CYP19), methadone also has been shown to directly inhibit CYP19 [37], potentially decreasing E2 concentrations further.

We found that lower E2 and lower free T concentrations were both independently related to lower LS BMD T-score, and, when we included these variables in the multivariable regression model, the effect of methadone maintenance therapy on LS BMD decreased by two-thirds and was no longer statistically significant. The relationship between sex hormones and BMD in men is complex with contributions from both androgen and estrogen receptor signaling [38, 39]. Consistent with a mediation effect of sex hormones on the relationship between methadone maintenance therapy and BMD, we observed that free T and E2 explained most of methadone’s effect at the LS, which is composed mostly of trabecular bone and is more susceptible to changes in the hormonal environment [40]. Whether T replacement therapy improves BMD or reduces fracture risk in men receiving methadone who also have hypogonadal symptoms and biochemical evidence of hypogonadism deserves further study.

Our study had multiple strengths. We conducted this study in the ALIVE cohort, a population of individuals who inject drugs, which is a hard-to-reach population with scarce data. We focused on an older population of individuals with opiate use disorder in a community-based, urban setting, who are at risk of aging-related comorbidities, such as osteoporosis. We used state-of-the-art liquid chromatography/MS assays to measure total T and E2. We relied on free T measurement using equilibrium dialysis, given the high prevalence of hepatitis C and HIV in our cohort and the SHBG abnormalities associated with these chronic infections. All our results were adjusted for these conditions to avoid confounding.

We also acknowledge the limitations of our study. First, by using a cross-sectional study design, causality could not be established, and the relationship between methadone, BMD, and sex hormones should be interpreted with caution. Second, although we performed mediation analyses using the cross-sectional data, the results will need to be interpreted with caution given the potential for reverse causality. However, our data are consistent with previous studies [4, 22‐24] and provide further evidence that the difference of LS BMD by methadone used is largely explained by level of sex hormones in our comprehensive evaluations. Third, compared to men, the sample size for women was relatively small. Therefore, we may not have sufficient power to evaluate the differences in BMD by methadone use among women. This also precluded our ability to assess the role of menopause and sex hormone in the association. Finally, we used BMD as an estimation of BMD, so further investigation is required to understand the effect of methadone, as well as other factors (eg, nutrition, physical exercise), on aging-related changes in BMD and the risk of fracture.

In conclusion, MMT usage may contribute to decreased LS BMD in a population of men who inject drugs, which appears to be mediated by both T and E2. These results provide further evidence that methadone may have a significant effect on BMD in a population of individuals with opiate use disorder. Further studies on the effect of T replacement or use of alternative opioid treatment (ie, buprenorphine) on BMD require further evaluation. These results have clinical implications suggesting that earlier, more aggressive DXA screening in individuals on long-term methadone therapy may be warranted to prevent fracture-related morbidity and mortality in this already at-risk population.

## Data Availability

Some or all data sets generated during and/or analyzed during the current study are not publicly available but are available from the corresponding author on reasonable request.

## Abbreviations

ALIVE: AIDS Linked to the Intravenous Experience
BMD: bone mineral density
BMI: body mass index
CV: coefficient of variation
DXA: dual-energy x-ray absorptiometry
E2: estradiol
FN: femoral neck
HCV: hepatitis C virus
IQR: interquartile range
LS: lumbar spine
MMT: methadone maintenance treatment
MS: mass spectrometry
OR: odds ratio
OUD: opioid use disorder
SHBG: sex hormone–binding globulin
T: testosterone
TH: total hip
